# Infected open depressed skull fracture complicated with tetanus grade I in an unimmunized child: a rare case report with literature review

**DOI:** 10.1186/s12245-021-00346-9

**Published:** 2021-04-23

**Authors:** Dzulfikar D. L. Hakim, Ahmad Faried, Adila Nurhadiya, Ericko H. Laymena, Muhammad Z. Arifin, Akhmad Imron, Iwan Abdulrachman

**Affiliations:** 1 Department of Child Health, Faculty of Medicine, Universitas Padjadjaran - Dr Hasan Sadikin Hospital, Bandung, Indonesia; 2 Department of Neurosurgery, Faculty of Medicine, Universitas Padjadjaran - Dr Hasan Sadikin Hospital, Bandung, Indonesia; 3 Department of Anesthesiology and Intensive Care, Faculty of Medicine, Universitas Padjadjaran - Dr Hasan Sadikin Hospital, Bandung, Indonesia

**Keywords:** Tetanus, Trismus, Lockjaw, Depressed skull fracture, Perioperative

## Abstract

**Background:**

Tetanus is a rare disease caused by *Clostridium tetani*, which produces tetanolysin and tetanospasmin. In 2018, there were only approximately ten tetanus cases reported in Indonesia. Despite widespread vaccination, especially in low–middle-income countries, tetanus still occurs (mostly in adults) due to the lack of immunization related to religious tenets, cultural belief, or inaccessibility to medical care. In addition, tetanus in the pediatric population shows features which are quite distinct from the adult group.

**Case presentation:**

We report a case of a 7-year-old girl presented to our institution with a history of falling 10 days prior to admission, with only skin laceration on her forehead. For 1 day prior to admission, the patient looked drowsy and difficult to be awakened, accompanied with stiffness of her jaw; we diagnosed her as an unimmunized child with an open depressed skull fracture of her frontal bone and wound infection complicated with “lockjaw.” Perioperative management of this rare case is reported and discussed.

**Conclusion:**

The pediatric intensive care of such patients requires halting further toxin production, neutralization of circulating toxin, and control of the clinical manifestation induced by the toxin that has already gained access to the central nervous system. The basic tenets of anesthetic care in such case must be well-managed and planned prior to surgery.

## Introduction

Tetanus is an infection that involves the nervous system, caused by a bacterium known as *Clostridium tetani* (*C*. *tetani*). It is different from other vaccine-preventable diseases because it does not spread from person to person. Spores of tetanus bacteria are everywhere in the environment and enters the body through damaged skin. The spores develop to become bacteria when they enter the body usually due to cuts or puncture wounds caused by contaminated objects. Tetanolysin damages surrounding tissue and is also capable of hemolysis. Although tetanolysin plays no direct role in the clinical manifestations of tetanus, it is thought to optimize the milieu for bacterial proliferation. Tetanospasmin (metalloprotease) produced by this bacterium travels through axons in a retrograde fashion to CNS leads to unopposed muscle contraction and spasm. Seizures may occur and the autonomic nervous system may also be affected. This is a preventable disease with available vaccinations. Nearly all tetanus cases are among people who did not get the recommended tetanus vaccination, which include people who have never received a tetanus vaccine and adults who do not have their 10-year booster shots. Although prevention has been incorporated in our national immunization program by the Indonesia Health Ministry, tetanus remains one of our major health problems. The World Health Organization reported that there were 391 cases of tetanus in children and neonates in Indonesia in 2019 [1, 2]. In this study, we reported a case report of a 7-year-old girl with inadequate immunization status that had a head injury and initially received suboptimal wound care, which led to tetanus and performed craniotomy debridement. Herein, we discussed the clinical presentation and management of the patient according to the literature.

## Case report

A 7-year-old girl was admitted to our hospital with decreased consciousness. Ten days prior to admission, when the patient was playing at a public restroom, she lost her balance and fell over with her head hit the floor that was made of cement. She had a skin laceration at her forehead; she was brought to a nearby primary healthcare facility, underwent wound suturing, and discharged with uneventful symptoms. Five days afterwards, she had a fever and purulent discharge from the wound; she was brought to the local hospital, underwent wound cleansing, and then discharged home. One day prior to admission, the patient looked drowsy and difficult to be awakened, accompanied with stiffness of her jaw; she was brought to another hospital. She received anti-seizure medication intravenously and then referred to our hospital.

**Fig. 1:**
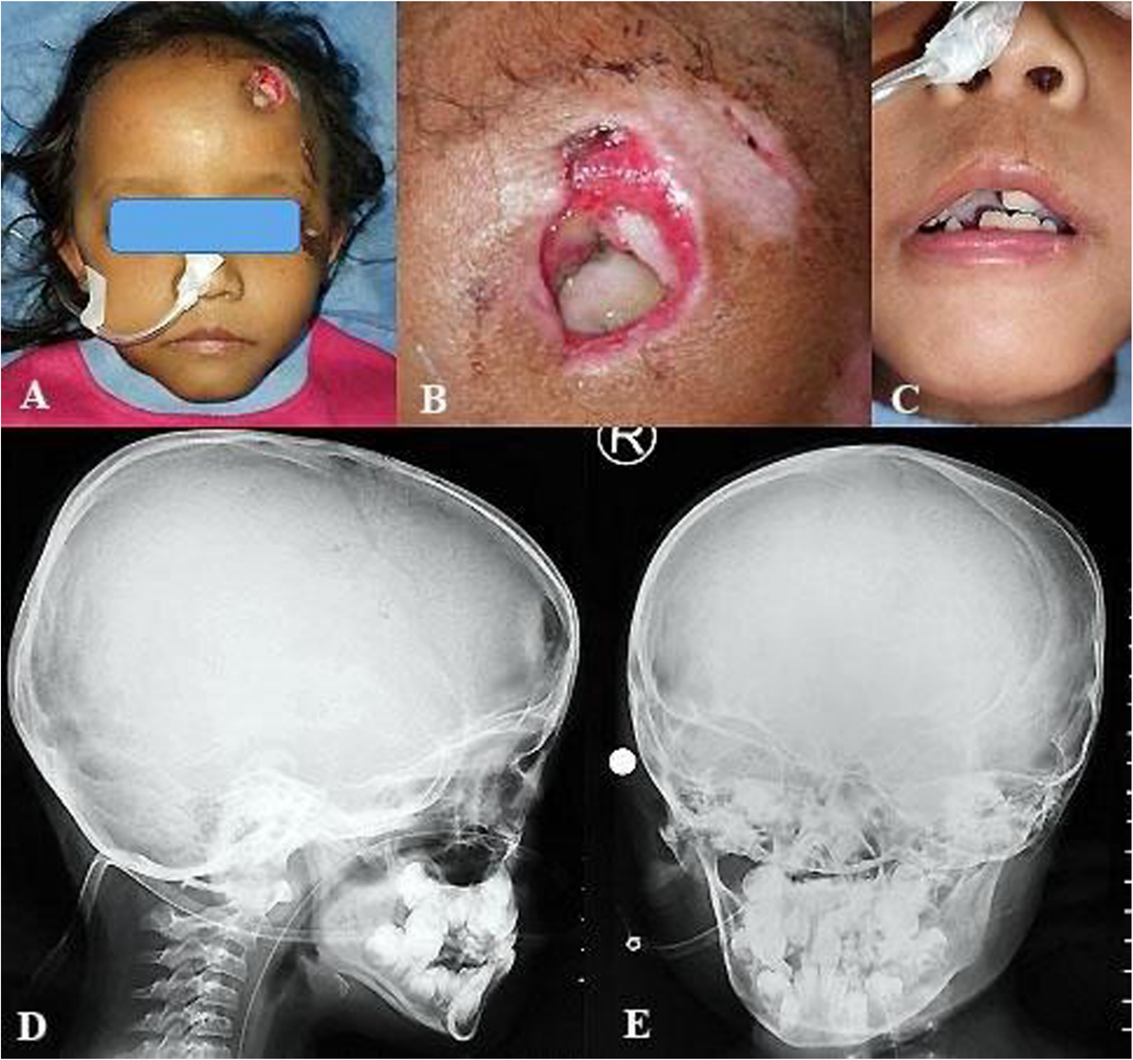
Representative images of patient condition. **a** There was a purulent wound at her left forehead. **b** Higher magnification, showing the condition of her wound. **c** She had a trismus with less than 1.5 cm breadth. **d**, **e** Skull X-ray demonstrating a double contour appearance at left the frontal scalp

**Fig. 2:**
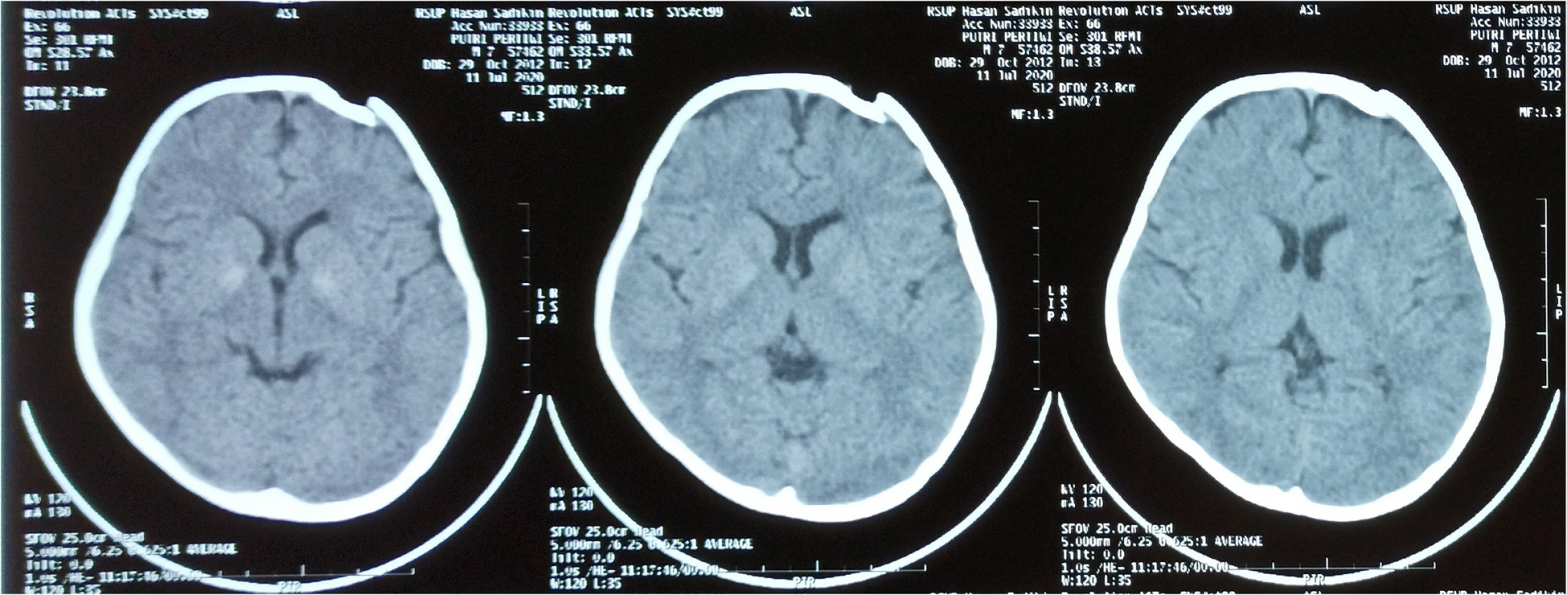
Contrast head CT scan which showed a depressed skull fracture of the left frontal bone

The patient had never received any vaccination since birth because the midwife was uncertain about administering vaccinations to low-birth-body-weight babies. Another issue was that due to some religious view, her family also rejected any vaccination for her. At admission, her vital signs were within normal limits. There was a purulent wound at her left frontal scalp, 2 × 1.5 × 0.5 cm in size, and on palpation, there was bone discontinuity; from neurological examination, she exhibited nuchal rigidity, trismus > 1 fingerbreadth, and motoric spasticity. Other tetanus signs such as spontaneous spasm, provoked spasm, board-like abdomen, and opisthotonos were absent. There was no autonomic dysfunction; laboratory results were mainly unremarkable, except for thrombocytosis (718 × 10^9^ cells/L). Skull x-ray demonstrates a double counter appearance at her left frontal scalp (Fig. 1). Head CT demonstrates a depressed skull fracture (Fig. 2).

The patient was diagnosed with an open depressed skull fracture and tetanus grade I based on clinical signs and symptoms, with the first sign being lockjaw followed by decrease of consciousness. For the tetanus treatment, the patient was given human tetanus immunoglobulin (HTIG) 3000 IU, ampicillin sulbactam 4 × 1 g IV, metronidazole 3 × 200 mg IV, and diazepam 4 × 2 mg IV. For the depressed skull fracture, she underwent craniectomy and debridement. The patient underwent general anesthesia and endotracheal intubation with a 5ID cuffed PVC endotracheal tube. The patient was premedicated with 60 μg of fentanyl, while the induction was performed with 50 mg of propofol and 20 mg of rocuronium. Throughout the operation, the patient was ventilated with O_2_ plus N_2_O (50:50) and 2% sevoflurane. The “lockjaw” trismus made intubating attempt to the patient challenging; hence, an extra dose of rocuronium (1 mg/kgBW) was given.

**Fig. 3:**
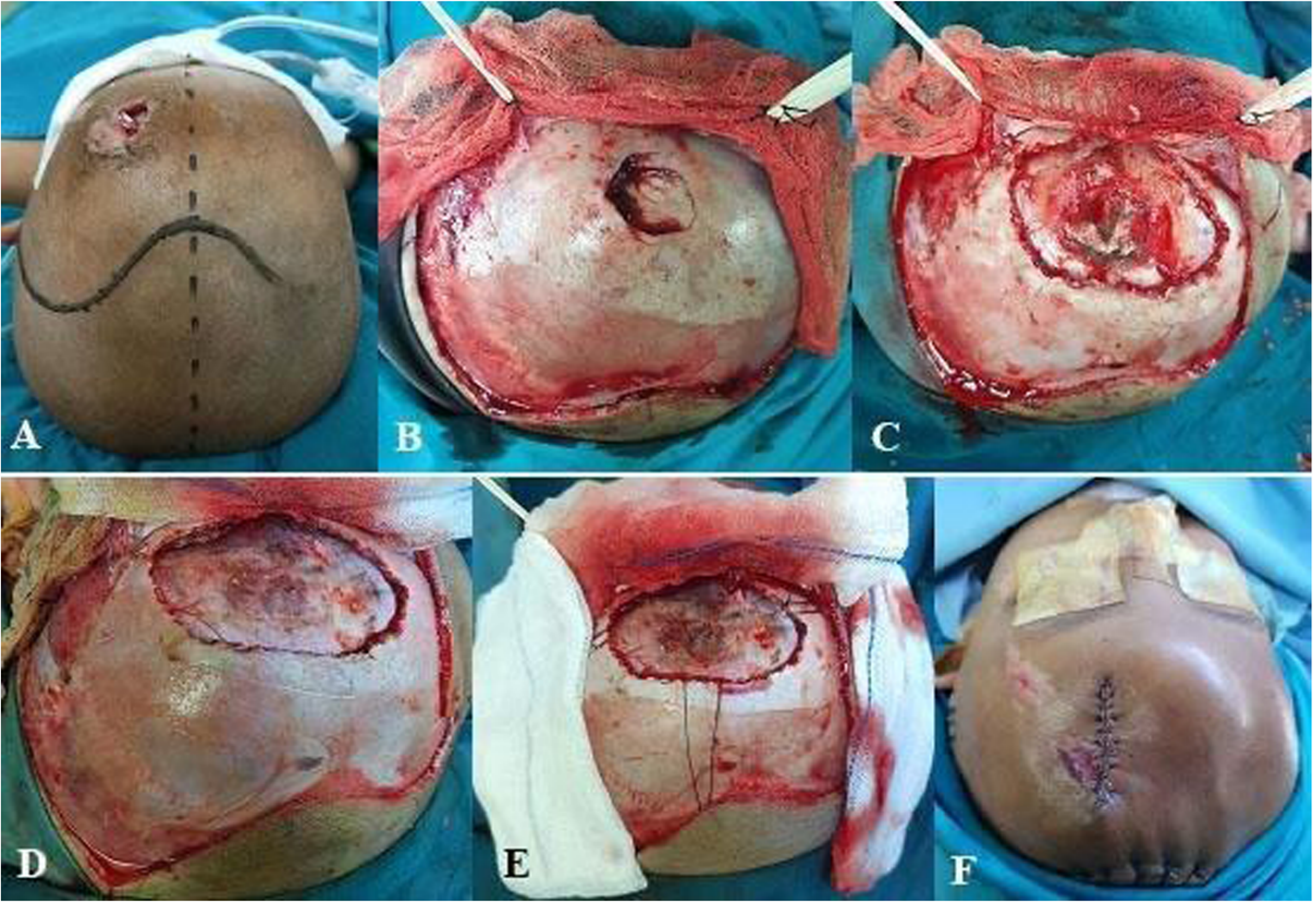
**a** The patient was positioned supine, and ¾ coronal incision was performed. **b** A depressed interlocking skull fracture about 3 × 2 cm. **c** Craniectomy was performed; the dura mater was yellowish, intact, and not tense. **d** Debridement was conducted. **e** Bone defect was not repaired. **f** The wound and incisional wound was primarily sutured

Intraoperatively, we found a fragmented interlocking fracture, with a dimension of 3 × 2 cm. Then, craniectomy was performed with boundaries of healthy bone. The dura mater was yellowish, intact, and not tense. The bone defect was not reconstructed, but rather the skin closed (Fig. 3). Pus was then sampled from the fracture location and sent for staining examination and culture. However, both examinations showed no microorganism growth. Postoperatively, the patient was sent to the pediatric intensive care unit for 3 days with mechanical ventilation and then transferred to the ward. After 3 weeks post-operative, the patient showed remarkable clinical recovery. After being discharged from the hospital, the patient was recommended to get the tetanus toxoid vaccine and other immunizations in primary health care.

## Discussion

Tetanus is an acute disease affecting the CNS. Each year, approximately 2.8 million people sustain head injuries in the USA alone, resulting in approximately 2.5 million emergency evaluations, 300,000 hospital admissions, and 60,000 deaths. The most common mechanism of injury was fall at 48%, followed by motor vehicle collision at 34%, and assault or being struck in the head at 30%. The most common location of injury reported was on the road (39%), followed by inside a home (21%) [3, 4]. There are several factors in our society that play role in the incidence of tetanus. There are still some religious tenets, cultural beliefs, and traditional healers that act as barriers to immunization and good wound care. In this case, the barrier to immunization was the midwife’s uncertainty in administering vaccination to a low-birth-body-weight baby, which the mother passively accepted. This is the first reported case tetanus complicating an untreated mild open head injury in Indonesia that underwent craniotomy debridement for source control [4].

Tetanus primary manifestation is prolonged muscular spasm (without altered mental status) caused by neurotoxin from *C*. *tetani* that is potentially fatal. *C*. *tetani*, a Gram-positive anaerobic bacterium, exists as a sporulated form in the environment (usually in soil) throughout the world and is found in the gastrointestinal tracts of animals and humans. *C*. *tetani* spores enter the body through a wound or damaged skin; in the presence of anaerobic conditions, the spores germinate. The bacteria produce two very potent toxins (tetanolysin and tetanospasmin). Both toxins enter the blood stream and lymphatic system to disseminate through the body. Tetanolysin is thought to optimize conditions for bacterial proliferation. The clinical features of tetanus are caused by tetanospasmin, which enters the peripheral nervous system directly from the contaminated wound, and is capable of affecting motor, sensory, and autonomic neurons. In approximately 20% of cases, no entry wound is noted. The primary pathological effect of tetanospasmin is the cleavage of synaptobrevin, which is a presynaptic protein. Synaptobrevin facilitates neurotransmitter fusion of vesicles to nerve membranes and release of their contents into the synapse. By cleaving synaptobrevin, neurotransmission is effectively blocked. Toxins act at several sites within CNS, including the peripheral motor end plates, spinal cord, and brain, as well as in the sympathetic nervous system. They cause the typical tetanus clinical manifestations by interfering with the release of neurotransmitters and blocking inhibitor impulses. This leads to unopposed muscle contraction and spasm. Seizures may occur and the autonomic nervous system also is affected [1, 5, 6].

After inoculation, the incubation period ranges from 3 to 21 days, averaging about 10 days. In general, the further the injury site is from the central nervous system, the longer the incubation period. A shorter incubation period is associated with more severe disease, more complications, and a higher mortality. Diagnosis of tetanus is based on historical and clinical signs and symptoms, so identification can be challenging for clinicians who may have never met a single case over the course of a career. Toxigenic of *C*. *tetani* can be cultured from wounds in patients but its presence is only supportive of the diagnosis, because it can be cultured from wounds in patients without tetanus [7]. Clinicians rarely recover the organism from the site of infection. The characteristic symptoms of tetanus are painful muscular contractions, primarily of the masseter and neck muscles and secondarily of trunk muscles. Trismus or “lockjaw” is a common and early sign of tetanus. Generalized spasms occur frequently, induced by sensory stimuli. History of an injury or apparent portal of entry may be lacking [1, 5, 6, 8].

There are four clinical feature types associated with tetanus: neonatal, generalized, localized, and cephalic; four grades of tetanus as proposed in the Ablett classification of tetanus severity are as follows: I (mild), II (moderate), III (severe), and IV (very severe) as we can see in Table 1 [7]. Neonatal tetanus occurs 3–7 days post-delivery. Difficulty feeding, poor suck/swallow, and excessive crying often precede overt spasms. Generalized tetanus accounting for > 80% of cases. The most common initial sign is spasm of the muscles of the jaw or “lockjaw” followed by painful spasms in other muscle groups in the neck, trunk, extremities, and generalized, seizure-like activity or convulsions in severe cases. Even with modern intensive care, generalized tetanus is associated with death rates of 10–20%. Localized tetanus is an unusual feature with consisting of muscle spasms in a confined area close to the site of the injury. Although localized tetanus often occurs in people with partial immunity and is usually mild, progression to generalized tetanus can occur. Cephalic tetanus is the rarest feature associated with lesions of the head or face and may also be associated with otitis media. The incubation period is short, 1–2 days. Unlike other types, cephalic tetanus results in flaccid cranial nerve palsies rather than spasm, but spasm of the jaw muscles may also be present. Like localized tetanus, cephalic tetanus can progress to the generalized form. Most cases of cephalic tetanus involve only the facial region below the eyebrows in CN V (trigeminal nerve) territory, in the periorbital region. Few cases attributable to the head injury usually were secondary to small scalp lacerations of the frontal temporal regions as a result of minor trauma. Cases secondary to open depressed skull fracture, such as our case, are almost unknown [6–19]. A study from Ethiopia demonstrated that trauma to be the most common portal of entry in children. This can be explained by the high chance of ignoring pediatric trauma and lack of provision of tetanus prophylaxis. The most common cause of death was the respiratory failure secondary to uncontrolled spasm (laryngospasm and diaphragm spasm); this may have been due to poor escalation of muscle relaxants and lack of pediatric intensive care unit (ICU) care [1, 7, 20, 21].

**Table 1:** Ablett classification of tetanus severity [7]

Grade	Clinical manifestation
I (mild)	Mild to moderate trismus Generalized spasticity No respiratory compromise No spasms Little or no dysphagia
II (moderate)	Moderate trismus Marked rigidity Mild to moderate but short spasms Moderate respiratory compromise with an increased respiratory rate (> 30 breaths per min) Mild dysphagia
III (severe)	Severe trismus Generalized spasticity Reflex prolonged spasms Increased respiratory rate (> 40 breaths per min) apneic spells Severe dysphagia Tachycardia (> 120 beats per min)
IV (very severe)	Clinical features of grade 3 tetanus Violent autonomic disturbances involving the cardiovascular system Severe hypertension and tachycardia alternating with relative hypotension and bradycardia (either of which might be persistent)

**Fig. 4:**
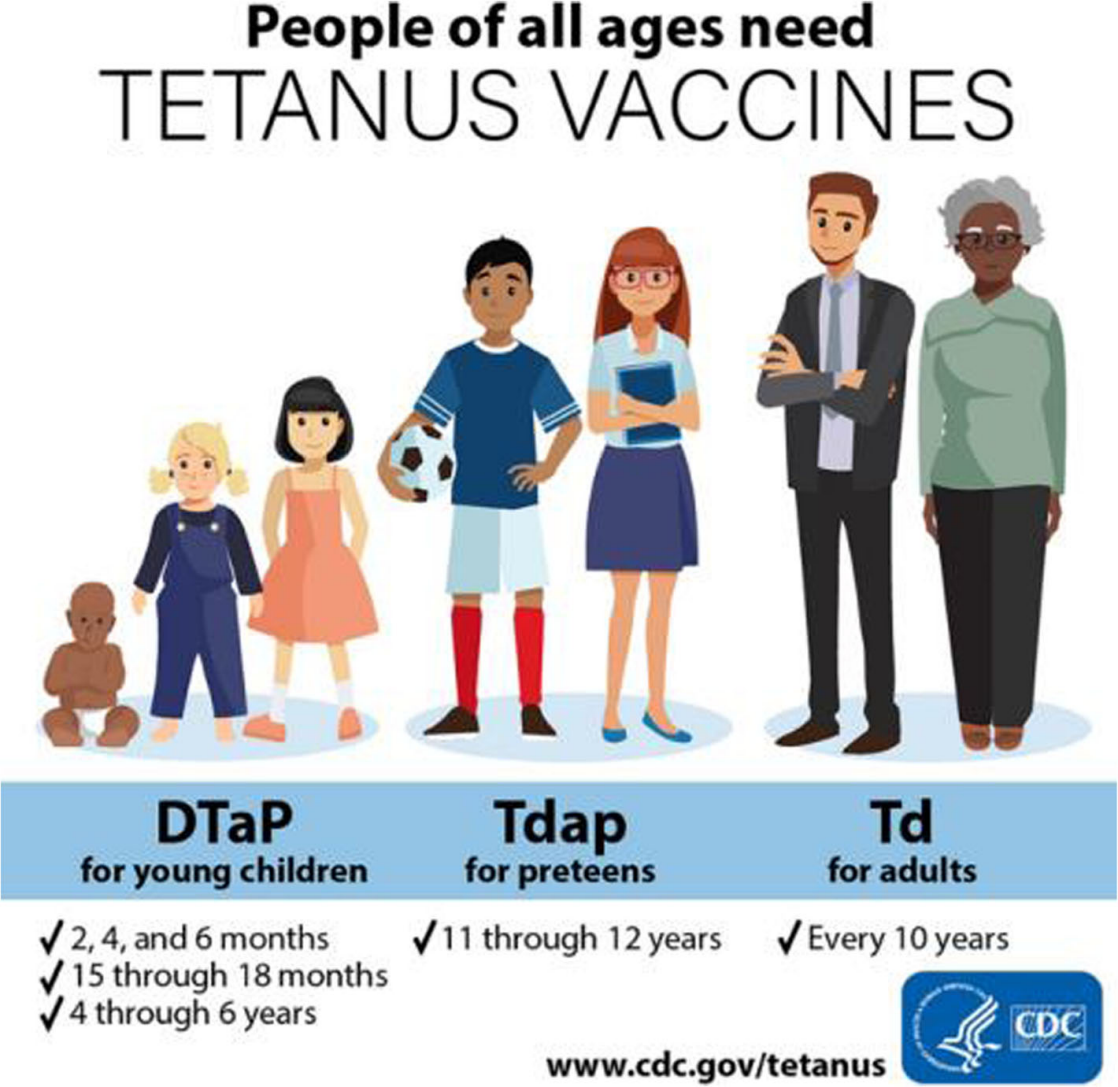
Graphic image icon highlights the CDC’s tetanus vaccination recommendations for young children, preteens, and adults

Tetanus is never completely eradicated because the bacteria maintain natural reservoirs in soil, humans, and other animals. Furthermore, there is no naturally acquired immunity; the very small amount of toxin necessary to cause disease does not stimulate antibody production in the host. Tetanus is almost entirely vaccine preventable since the vaccine efficacy is virtually 100%. A 6-dose series of tetanus toxoid-containing vaccine (TTCV) has been shown to provide immunity and protect women through their childbearing years, though some countries still endorse a 5-dose series (shown in Fig. 4). Indonesia’s national immunization programs include 5-dose TT vaccination. Three doses were given before the first year after birth, boostered at 4–7 and 15 years old. Vaccination and good wound care are the mainstays to prevent tetanus [5, 22].

The treatment of tetanus involves providing supportive care for symptoms of muscle spasms and potential respiratory compromise, neutralizing the remaining tetanus toxin, and good wound care for eradicating bacteria at the wound site. The defining factor that contributes to therapeutic success was time for immunoglobulin administration to neutralize the toxin and/or tetanus toxoid vaccination. Without treatment, the case fatality rate remains ~ 100%; with treatment, case fatality rates drop to 10–20%. For generalized tetanus, intensive care, including endotracheal intubation, mechanical ventilation, deep sedation, and/or paralysis, is the mainstay of supportive care. Patients should have minimal environmental stimulation to avoid the reflex spasms. Benzodiazepines, particularly diazepam, are treatments of choice, since they provide sedation, control muscle spasm, and provide anxiolysis. Intravenous magnesium sulfate can be used as an adjunct anti-spasmodic agent that also decreases autonomic instability. Intrathecal baclofen has shown promising results for severe spasms but may not be feasible in resource-poor areas where intrathecal catheter insertion is not practical and mechanical ventilator support is unavailable; higher dose cause respiratory depression and cardiovascular instability. Autonomic instability can be treated with clonidine, b-blockers, and morphine [5, 23, 24].

Rapid sequence intubation is mandated due to significant aspiration risk because of increased abdominal pressure, gastric stasis, and involvement of laryngeal muscles in tetanus patient. Preparation for a potentially difficult airway was in place, and spontaneous ventilation was maintained by the induction of anesthesia with sevoflurane in 100% O_2_. Various intravenous and inhalation anesthetic agents have been used without incidence in tetanus patients [25]. In our case, there were several risk factors related to tetanus complications. Firstly, she never received any vaccination and the wound was not properly taken care of; moreover, she did not get immunoglobulin to neutralize the toxin after the accident.

The pediatric intensive care of such cases requires halting further toxin production, neutralization of circulating toxin, and control of the clinical manifestation induced by the toxin that has already gained access to the CNS. The basic tents of anesthetic care in a patient with tetanus include consideration of the implication of a full stomach, prevention of the paroxysm of muscle spasm by maintaining a deep plane of anesthesia or the use of regional anesthesia and control of the autonomic instability. As the autonomic instability may be life-threatening, invasive arterial blood pressure monitoring may be indicated. Although uncommon, with prolonged tetanus, associated cardiomyopathy repeated exposure to catecholamines may be present. A perioperative assessment of renal and electrolyte balance required the presence of myoglobinuria and renal dysfunction [21, 25]. In our case, 3 weeks postoperative course was uneventful, and the patient showed an excellent clinical recovery and discharged on postoperative day 16.

## Conclusion

In summary, we present a rare case of a 7-year-old unimmunized girl with open depressed skull, fracture of frontal bone, and wound infection complicated with “lockjaw.” This case report highlights the crucial role that proper, optimal wound management and tetanus immunization in children have in preventing tetanus. Furthermore, this report also points out the need to improve the public awareness and knowledge of hygiene, sanitation, and the monumental benefits of immunization on one child’s life.

## Data Availability

Not applicable

## Abbreviations

CNS: Central nervous system
CT: Computed tomography
PVC: Polyvinyl chloride
CN: Central nervous
TT: Tetanus toxoid
